# Event and Comment

**Published:** 1915-11

**Authors:** 


### DENTAL REGISTER

Vol. LXX NOVEMBER, 1915 No. 11

### EVENT AND COMMENT

PROFESSOR CASSIDY QUITS TEACHING. A large number of his students will learn with regret that their beloved instructor in chemistry in the Ohio College of Dental Surgery has given up teaching. For forty-three years Professor Cassidy has taught chemistry in this Institution, which is a record of faithfulness to the cause of science that any man should be proud of. We shall never forget the painstaking and courteous efforts he made to clear up this, to us, most difficult subject. We studied Fowne's Chemistry, which was really chemical physics, inorganic and organic chemistry combined, until we almost dreamed it. We were so anxious to make good that we fear other subjects were neglected because we wanted to please this teacher especially. He knew chemistry and could teach it better than the book, and he had the faculty of inspiring his students with the idea that chemistry was an important subject for a dentist to know. His earnestness was contagious, and he established sympathetic relations with his subject and his students that produced the highest results. If he had not loved his subject and his pupils with a devotion that is all too rare in teachers he could never have made the personal sacrifices he was required to make throughout these years in the long trips from his home to the college in all kinds of weather, especially since the usual motive of a monetary compensation was in his case wholly lacking. It was a labor of love with him. We presume it must be the necessities of advancing years that now compels him to give up such a splendid record of faithful devotion to the cause which he loved more than any other. Doubtless there will be some one to take up the work where he leaves it; but to his old pupils it can never be so well done by anyone else. His connection with the faculty of this school has been one of strength not alone as a devoted teacher, but as a business adviser he will be greatly missed. He was always an excellent business man, and a loyal professional gentleman, and his influence here will be missed in faculty councils, we are sure. As a writer and contributor to professional literature he has made an enviable record. His book on Dental Materia Medica and Chemistry is a classic. His contributions to Dental Societies in the way of papers and discussions are always given attention and command respectful hearing because of his carefulness and accuracy of statement. We trust he may find time for further literary contributions to our journals and society meetings now that the tediousness of teaching will give him opportunity for this kind of work. No one but a teacher perhaps will be able to estimate what a sacrifice Doctor Cassidy has made for his profession by his long and faithful work as a teacher. It is wonderful how interested one can become in this kind of work. It is not unlike the joys one experiences from seeing the development of a business, or the growth of the seed to the plant and its flowers or fruit, it interests all the way. It is this that keeps us at our job. If it interests us we learn to love it and give our very life to it if necessary. Professor Cassidy seems to us to have always loved his work, and therefore his devotion. We regret that he feels called upon to quit, but he can be assured that his work will not end because he has vacated the chair of Chemistry in the Ohio College of Dental Surgery.

DIRECTOR EASTMAN DISPENSARY. Dr. Harvey J. Burkhart of Batavia, N. Y., has just been selected as director of this newest dental institution. Everyone who knows Dr. Burkhart will cordially approve the wisdom of this selection. He has proved himself one of the best executives in the profession, and he is a thorough professional man with the broad sympathies. We know of no one more completely fitted for the great task of organizing this great work. The trustees have certainly acted most wisely in securing his services as it guarantees the successful inauguration of the work.

PANAMA PACIFIC DENTAL CONGRESS. This the sixth World's Dental Congress is now a matter of history, and although as an important event its activities are closed, its results in marking the progress of the profession in the entire dental world can not be satisfactorily inventoried at this time. Owing to the disturbed conditions in the old world, it can not be said that it was a cosmopolitan affair exactly, although quite a number of dentists from foreign countries attended its sessions. It is estimated that there were about 1,200 dentists in attendance, the large majority of whom were from the middle and western states. The meetings were well taken care of in a fine and commodious building, except there was some criticism as to the management on the part of those in charge of the section meetings. The local committee did all they could to take care of the large attendance and everything was done to make the occasion socially pleasant as well as professionally profitable. Like all such affairs, it is difficult for one to attend all the functions, and consequently one is bound to miss some of the things in which he is most interested. The program was most excellent, and while it will not be practicable for those who were not in attendance to derive the most advantage from published reports of the clinical and social features of the occasion, the printed proceedings will put on permanent record the more substantial contributions. In this connection a notice appears in this issue to the effect that these proceedings can be had as soon as printed on the payment of ten dollars to the Pacific Dental Congress Commission. We are informed the procedings, in part at least, are to be published in the *Items of Interest.* It seems that the advanced membership fees have not been sufficient to enable the committee to print the proceedings independently, but the entire proceedings and the souvenir program will be supplied to all subscribers who will remit the amount to the commission. Owing to the old world troubles it is likely that this will be the last great world congress for many years, and every one who has kept up with the proceedings of the former congresses will surely want the proceedings of this one. Therefore it is hoped that enough dentists will send the money to the commission to have the proceedings properly published for future preservation in book form.

The proceedings of the several other national bodies meeting at the same time will be published in their regular records. As the proceedings of these organizations were largely formal and subversive to the proceedings of The Congress, these are comparatively unimportant.

AMERICAN DENTAL SERVICE TO THE ALLIED ARMIES. In another part of this issue we are publishing the report of the Dental Department of the American Hospital for wounded soldiers in Paris. This work is entirely voluntary on the part of Drs. Geo. B. Hayes and W. S. Davenport, American dentists practicing in Paris. The report is interesting because it shows the great need of such services, and also because of the character of the service rendered and the care used to record for scientific data the results obtained. The spirit of devotion to suffering humanity, and the great sacrifice of time and money involved, are refreshing in these times of selfish aggrandizement, and we ought to be proud that it has come to two such noble men as Drs. Hayes and Davenport to represent American dentistry in this splendid manner. We do not know all the needs of this work, but we are sure such a cause needs money as well as men to carry it on successfully, and we trust our readers may be interested to contribute whatever they can to this cause. We know the men and can assure any one that every dollar will be used to the best advantage, and should any one desire to offer his services to the cause, we shall be glad to put him in the way of getting all needed information. It is a grand opportunity for a young man to get a most unusual and valuable experience.

THE MILLER MEMORIAL. Next month at the annual meeting of the Ohio State Dental Society the statue to commemorate the career of Dr. W. D. Miller will be dedicated at Columbus. It is to be placed in the grounds of the Ohio State University, which seems highly appropriate, as he was an Ohioan by birth, and as his career was so largely an educational one, it seems eminently fitting that it be placed in such an atmosphere. It has been a matter of widespread interest and co-operation to secure the necessary funds for honoring a man who did so much toward making our treatment of dental caries scientific rather than empiric. It has been a matter of profound regret the world over that he could not have lived to see realized his great hope that some way should be opened that would give to the poor immunity to the most prevalent disease known. His was a great heart as well as a great mind, and had he lived even to the present time he would have rejoiced to see the grand work that is being undertaken for suffering humanity. In this attempt to erect a memorial of permanence to Dr. Miller, the profession will not only mark an epoch of civilization, but record a prophecy of future happiness when the ravages of dental caries will be a matter of history rather than a menace to human health and happiness.

